# Supplementary material for: Artefact-removal algorithms for Fourier domain quantum optical coherence tomography
Source: Sci Rep. 2021 Sep 20;11:18585. doi: 10.1038/s41598-021-98106-5 (PMC8452642; doi:10.1038/s41598-021-98106-5)
Supplement: Supplementary file 1 — Supplementary Information. [file 41598_2021_98106_MOESM1_ESM.pdf]

Artefact-removal algorithms for Fourier domain Quantum Optical Coherence Tomography

Sylwia M. Kolenderska[1*†] and Maciej Szkulmowski[2†]**

[1]The Dodd-Walls Centre for Photonic and Quantum Technologies, Department of Physics, University of Auckland, Auckland 1010, New Zealand
[2]Institute of Physics, Faculty of Physics, Astronomy and Informatics, Nicolaus Copernicus University in Toruń, Grudziadzka 5, 87-100 Torun, Poland
[†]These authors contributed equally to this work
[*]skol745@aucklanduni.ac.nz
[**]maciej.szkulmowski@fizyka.umk.pl

Two-dimensional Fourier transformation of a rotated joint spectrum in Fd-Q-OCT

To see why the additional multiplication by $\sqrt{2}$ is necessary, we consider a two-dimensional Fourier transformation applied along the axes parallel, $\omega_{\parallel}$, and perpendicular, $\omega_{\perp}$, to the main diagonal. This operation is analogous to a rotation of the coordinate system by $\pi/4$. In such a case, the new coordinates are given by:

$$\begin{bmatrix}\omega_{\parallel}\\ \omega_{\perp}\end{bmatrix} = \frac{\sqrt{2}}{2}\begin{bmatrix}1 & -1\\ 1 & 1\end{bmatrix}\begin{bmatrix}\omega_{\alpha}\\ \omega_{\beta}\end{bmatrix} \tag{1}$$

where

$$\begin{aligned}\omega_{\parallel} &= \frac{\sqrt{2}}{2}(\omega_{\alpha}-\omega_{\beta}) \\ \omega_{\perp} &= \frac{\sqrt{2}}{2}(\omega_{\alpha}+\omega_{\beta})\end{aligned} \quad \text{and} \quad \begin{aligned}\omega_{\alpha} &= \frac{\sqrt{2}}{2}(\omega_{\perp}+\omega_{\parallel}) \\ \omega_{\beta} &= \frac{\sqrt{2}}{2}(\omega_{\perp}-\omega_{\parallel}).\end{aligned} \tag{2}$$

The term $P_{n,u}$ responsible for positioning the peaks in a two-dimensional Fourier transform can be rewritten:

$$\begin{aligned}P_{n,u}(\omega_{\alpha},\omega_{\beta}) &= \exp\Big(i\big(\tilde{z}_n\omega_{\alpha} - \tilde{z}_u\omega_{\beta}\big)\Big) = \\ &= \exp\Big(i\frac{\sqrt{2}}{2}\big(\tilde{z}_n(\omega_{\perp}+\omega_{\parallel}) - (\tilde{z}_u(\omega_{\perp}-\omega_{\parallel})\big)\Big) = \\ &= \exp\Big(i\sqrt{2}\big(\frac{\tilde{z}_n+\tilde{z}_u}{2}\omega_{\parallel} + \frac{\tilde{z}_n-\tilde{z}_u}{2}\omega_{\perp}\big)\Big) = \\ &= \exp\Big(i\sqrt{2}\bar{\tilde{z}}_{nu}\omega_{\parallel}\Big)\exp\Big(i\frac{\sqrt{2}}{2}\Delta\tilde{z}_{nu}\omega_{\perp}\Big) = \\ &= P_{n,u}(\omega_{\parallel},\omega_{\perp})\end{aligned} \tag{3}$$

$\omega_{\parallel}$ and $\omega_{\perp}$ are related to geometrical coordinates and need to be expressed in terms of the central frequency ω_0 and frequency detuning, ω', to truly represent the diagonals. Using the two first relationships of (2) and the fact that ω_{α} and ω_{β} represent negatively correlated photons, for which $\omega_{\alpha} = \omega_0 - \omega'$ and $\omega_{\beta} = \omega_0 + \omega'$, we obtain:

$$\begin{aligned}\omega_{\parallel} &= -\sqrt{2}\omega' \\ \omega_{\perp} &= \sqrt{2}\omega_0\end{aligned} \tag{4}$$

Using (4) in (3) gives:

$$P_{n,u}(\omega_{\parallel},\omega_{\perp}) = \exp\Big(-i2\bar{\tilde{z}}_{nu}\omega'\Big)\exp\Big(i\Delta\tilde{z}_{nu}\omega_0\Big) = P_{n,u}(\omega',\omega_0) \tag{5}$$

After two-dimensional Fourier transformation along ω' and ω_0 we obtain:

$$m(z_\parallel, z_\perp) = \mathscr{F}\left\{P_{n,u}(\omega', \omega_0)\right\} = \delta\left(z_\parallel + 2\bar{\tilde{z}}_{nu}, z_\perp - \Delta\tilde{z}_{nu}\right) \tag{6}$$

At the diagonal, for which $n = u$, $m(z_\parallel, z_\perp)$ simplifies to:

$$m_{diag}(z_\parallel, z_\perp) = \delta(z_\parallel + 2\tilde{z}_n, z_\perp) \tag{7}$$

It can be clearly seen from (7) that indeed only the components along the diagonal remain. Also, these remaining components represent peaks which are separated by twice the distance what leads to two-fold resolution increase.
